# Study on Shear Behavior and Failure Characteristics of Bolted Anisotropic Rock Joints

**DOI:** 10.3390/ma16062210

**Published:** 2023-03-09

**Authors:** Yujing Jiang, Xinpeng Li, Jiankang Liu, Hengjie Luan, Sunhao Zhang, Changsheng Wang, Dong Wang

**Affiliations:** 1 College of Energy and Mining Engineering, Shandong University of Science and Technology, Qingdao 266590, China; 2 State Key Laboratory of Mining Disaster Prevention and Control Co-Founded by Shandong Province and the Ministry of Science and Technology, Shandong University of Science and Technology, Qingdao 266590, China; 3 School of Engineering, Nagasaki University, Nagasaki 852-8521, Japan; 4 Inner Mongolia Shanghaimiao Mining Co., Ltd., Ordos 016299, China

**Keywords:** anisotropic rock joint, shear characteristics, breaking displacement, bolt deformation

## Abstract

Anisotropic discontinuity exists widely in rock masses of mines, tunnels, slopes, water conservancy and hydropower projects. The shear characteristics of bolted anisotropic rock joints are extremely important for the stability design of engineering rock mass. However, few scholars have studied the bolted anisotropic rock joint. The different rock properties on both sides of the rock joint, especially the different rock strengths, will greatly affect the deformation characteristics and failure mode of the rock mass. Based on this, a series of shear tests were carried out on the bolted anisotropic rock joint under different normal stresses, and the characteristics of shear stress–shear displacement curve, shear strength, failure characteristics of the rock joint and deformation characteristics of the bolt are discussed. λ is defined as the strength ratio of upper and lower rock on the structural surface. The results show that the effect of λ on the shear stress–shear displacement curve is not obvious at the pre-fracture stage. The shear stress–shear displacement curve at the pre-breaking stage of the bolt presents a softening stage when the normal stress is equal to 0.5 MPa, tends to be horizontal when the normal stress is equal to 1 MPa and presents a hardening stage when the normal stress is greater than 1 MPa. After the bolt is broken, the shear stress–shear displacement curve presents a stepped-down descent. With the increase in λ, the breaking shear stress of the bolt increases. Elliptic failure occurs on the surface of the bolted anisotropic rock joint, and the length of the major axis of the ellipse decreases with the increase in λ value and normal stress. The bolts with different λ values of anisotropic rock joint show “Z-shaped” tensile bending deformation characteristics after shear fracture, and the horizontal and vertical components of the bolt deformation decrease with the increase in λ value and normal stress. The fracture shear displacement of the bolt increases with the increase in normal stress and decreases with the increase in λ value. The research results are helpful to further understand the shear mechanical characteristics and differences of bolted rock joints and provide a reference for solving the engineering problems of the composite layered rock mass.

## 1. Introduction

There are a large number of rock joints in the rock mass of mines, tunnels, slopes, water conservancy and hydropower projects, which weaken the integrity and strength of the rock mass [1,2]. Many engineering practices have shown that rock joint shear slip is one of the key causes of rock mass engineering instability [3,4]. Full-length bolted support can effectively control shear slip deformation of the rock mass to realize stability control of rock mass, which has become one of the most widely used means in rock mass engineering reinforcement [5,6,7,8,9,10]. However, with increasingly complex engineering conditions, the failure of bolted support induced by rock joint shear slip becomes increasingly prominent. Under the combined load of tensile and shear, the bolt is more likely to lose the controllability of surrounding rock deformation and even lead to the failure of the whole supporting system and induce a large deformation disaster of surrounding rock [11,12]. Therefore, it is very important to study the shear characteristics of bolted rock joints for the stability evaluation and control of rock mass engineering.

At present, experts and scholars at home and abroad have carried out many laboratory tests on the shear characteristics of bolted rock joints. Jiang et al. [13] carried out a series of shear tests on rough joints of bolted and unanchored rock materials under different constant normal stiffness (CNS) boundary conditions and studied the effects of CNS boundary conditions on shear mechanical properties of bolted joints, shear failure of the joint, shear deformation of the anchor bolt and shear displacement characteristics of the anchor bolt. Srivastava et al. [14] conducted direct shear tests on large-size specimens to study the effects of bolt spacing and bolted area on their bolted performance. Wang et al. [15] studied the characteristics of the shear mechanical properties, the failure mechanism and the acoustic emission parameters of the anchored joints are studied under different surface roughnesses and anchorage conditions. Based on the laboratory direct shear test, Zheng et al. [16] studied the influence of two locking modes, two-end locking and no-locking, on the shear characteristics of bolted joints and analyzed the enhancement effects of normal stress and prestress on the shear strength of bolted joints. Spang et al. [17] studied the influence of bolt parameters and joint parameters on shear strength by conducting indoor shear tests. Liu et al. [18] carried out double-sided shear tests on frictionless bolted joints. By comparing the shear strength of the bolted joint under different bolted angles, the influence of bolted angle on the shear performance of the bolted joint was analyzed, and the influence of bolted angle on the shear action of the bolted joint was further clarified.

The above studies reveal the influence law of shear stiffness, normal stress, bolted angle and bolt preload on the shear characteristics of the bolted rock joint and have important significance for understanding the shear characteristics of the bolted rock joint. It is worth noting that the above research results are concentrated in the case of the same rock properties on both sides of the rock joint, while there are few studies on the bolted anisotropic rock joint. Furthermore, anisotropic rock joints are widely developed, among which the interbedded sandstone and mudstone in the Three Gorges Reservoir area of China is a typical representative of the anisotropic rock joints [19]. The different rock properties on both sides of the rock joint, especially the different rock strengths, will greatly affect the deformation characteristics and failure mode of the rock mass. On the one hand, many studies have shown that rock strength has a significant impact on the shear characteristics of the bolted rock joint. For example, He et al. [11] conducted the bolted rock joint shear test of three rock strengths and found that the shear breaking displacement and shear energy absorption of the bolt decreased with the increase in the strength of the surrounding rock. Yang et al. [20] studied the influence of rock bolts in rock joints with surrounding rock strength and found that increasing the compressive strength of surrounding rock can reduce the axial force of rock bolts with the growth rate of shear. On the other hand, different rock strength combinations can also affect the shear characteristics of the discontinuity itself. For example, He et al. [21] proposed the shear strength criterion of the anisotropic discontinuity of rock mass in 1994. Fang et al. [22] took the anisotropic layer of typical silty mudstone and marl of Badong Formation as the research object, conducted direct shear numerical tests based on particle flow software, and found that the shear failure of the anisotropic joint mainly evolved uniformly and synchronously along the soft rock side of the contact surface. The coupling between the rock mass and the bolt is the key to the support effect of the bolt. Especially when the strength of rock mass on both sides of the rock joint is different, due to the strength difference of rock mass, the bolt will produce asymmetric deformation along the rock joint, and the support effect of the bolt will change significantly. Therefore, the influence of different rock strength combinations on the shear behavior of bolted rock joints cannot be ignored.

In view of the above knowledge, this paper reveals the shear characteristics, failure and deformation characteristics of the rock joint and bolt by preparing the bolted anisotropic rock joint with different wall rock strengths and through laboratory test studies. The research results are helpful to further understand the shear mechanical characteristics and differences of the bolted rock joint and provide a reference for solving the engineering problems of the composite layered rock mass.

## 2. Laboratory Direct Shear Test

### 2.1. Specimen Preparation

**Figure 1:**
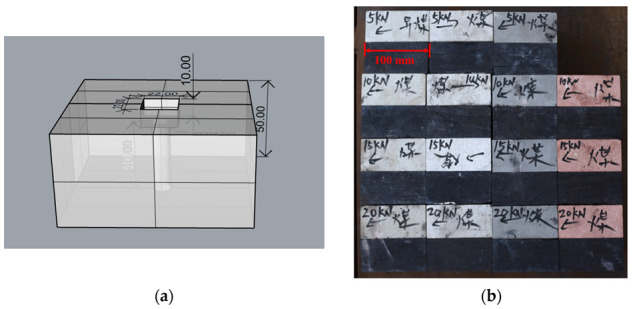

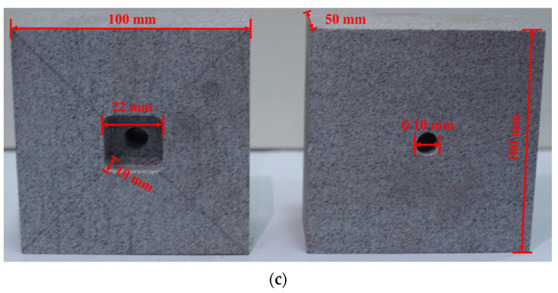
Specimen material drawing. (**a**) Three-dimensional transparent diagram (Unit: mm); (**b**) Test pieces of different anisotropic rock joints; (**c**) Single test piece.

To study the shear characteristics of anisotropic anchored rock joints, a large number of anchored anisotropic rock joints were prepared, and direct shear tests were carried out. The coal specimens required for the test were taken from Tangkou Coal mine in Jining City, Shandong Province, and other rock specimens were taken from protolith sandstone specimens of different strengths from a stone yard in Shandong Province. The sandstone specimens were divided into red sandstone, black sandstone, green sandstone and white sandstone according to different colors. Each sandstone has a different strength [23]. All the rocks were processed into cuboid specimens with length, width and height of 100 mm × 100 mm × 50 mm, as shown in Figure 1. A hole with a diameter of 10 mm was left in the middle of the specimen to place the anchor rod. The anchor bolt used in the test was 8.8-grade carbon steel, and each end was 10 mm beyond the groove of the specimen to install the nut and gasket. The anchorage agent used is SKO-modified epoxy planted plastic, which is often used for bolting and supporting various equipment foundations and roof walls of roadways in mines. This anchoring agent has aging resistance, freeze–thaw resistance, long-term resistance, fatigue resistance, medium resistance and corrosion resistance. The specific parameters of the bolt and anchoring agent are shown in Table 1.

**Figure 2:**
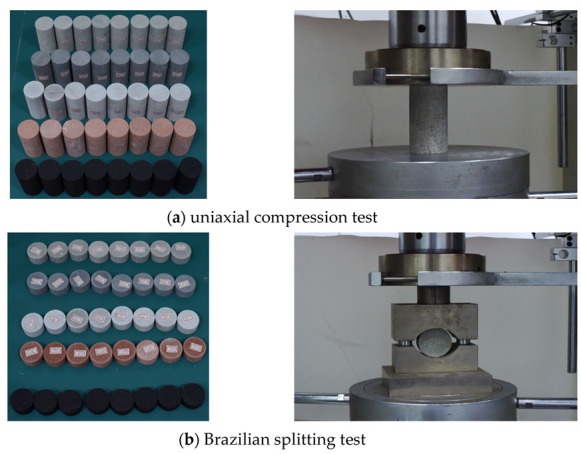
Test of mechanical properties.

Before the direct shear test, a large number of rock uniaxial compression with a height of 100 mm and a diameter of 50 mm and Brazilian splitting standard specimens with a thickness of 25 mm and a diameter of 50 mm were also prepared to test the mechanical properties of rock strength, as shown in Figure 2. The main mechanical parameters are shown in the following Table 2.

**Figure 3:**
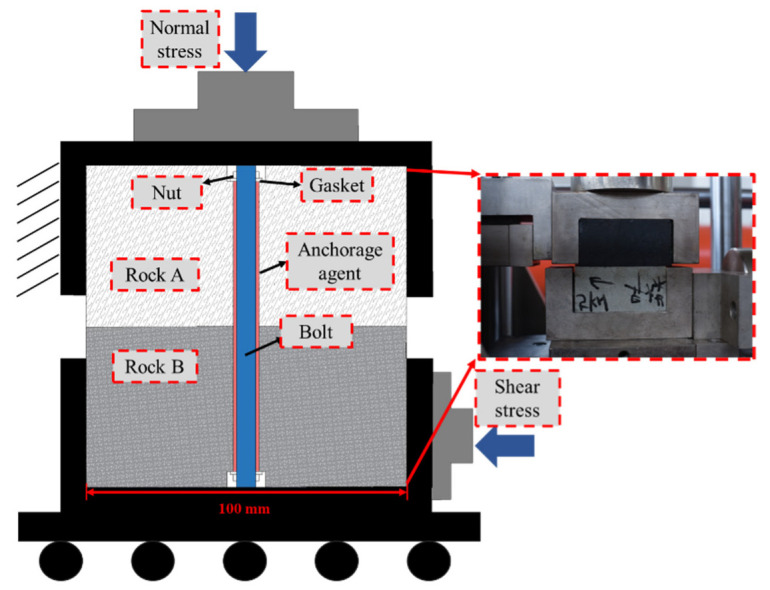
Shear test model diagram.

During the preparation of the specimen, the rock-fixing footwall was coal, and the hanging wall was matched with different rocks. First, the anchorage agent was injected into the reserved holes, and then the bolt was passed through the holes and fixed with nut brackets at both ends to construct the opposite rock joint. After the preparation of the specimen, it was solidified at 20 °C for 48 h to ensure its maximum strength. The final specimen size is 100 × 100 × 100 mm. The shear test model of the bolted anisotropic rock joint is shown in Figure 3.

### 2.2. Testing Equipment

**Figure 4:**
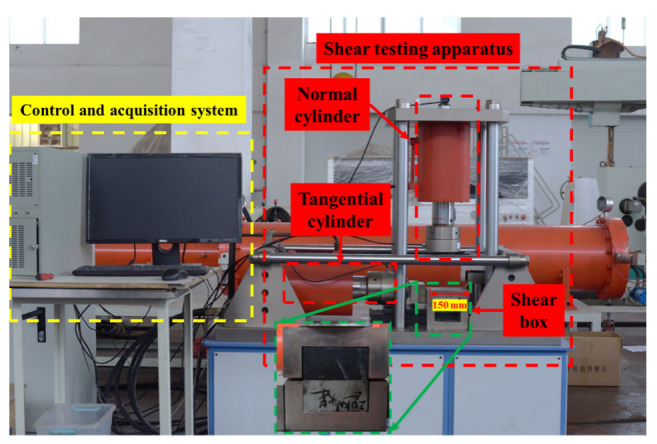
Test equipment diagram.

The RJST-616 rock joint shear tester [24], as shown in Figure 4, was used to carry out the shear test. The normal force and shear force of the shear test system were provided by the normal and tangential cylinders, respectively. The maximum output of the vertical hydraulic cylinder was 1000 kN, and the piston stroke was 50 mm. The maximum output of the horizontal hydraulic cylinder is 500 kN, and the piston stroke is 50 mm. The deformation rate is 0.0001~1 mm/s. During the shearing process, the upper part of the shearing box is fixed, while the lower part moves incorrectly along the shearing direction. After the sample was placed in the shear box, the normal stress was first applied to a fixed value in the normal direction according to the force-controlled loading mode, and then the shear force was applied in the tangential direction according to the displacement-controlled loading mode of 0.5 mm/min. The test was stopped after the bolt was broken in the shear process, that is, after the obvious fall on the shear stress–shear displacement curve entered the flatness stage. Constant normal stress boundary conditions are adopted in the test. Considering that common anchorage engineering support is often found in slope management, underground roadway and chamber within a shallow range of 2~3 m, and the shallow surrounding rock breakage causes the high stress to transfer to the deep, the surrounding rock stress within the support range is far lower than the original in situ stress [13]. According to relevant experience [16], The normal stresses applied in this test are 0.5, 1, 1.5 and 2 MPa, respectively.

## 3. Test Results and Analysis

The difference between the bolted anisotropic rock joint and the bolted rock joint lies in the difference between the rocks on both sides of the rock joint, and the first is the difference in strength. In order to reflect the difference in strength between the two sides of the rock joint wall rock, λ is defined to characterize the strength ratio of the rock on the rock joint and the footwall coal. Among the rock combinations, λ = 2.35 coal-red sandstone, λ = 3.78 coal-black sandstone, λ = 4.16 coal-blue sandstone and λ = 6.72 coal-white sandstone.

### 3.1. Shear Stress–Shear Displacement Analysis

**Figure 5:**
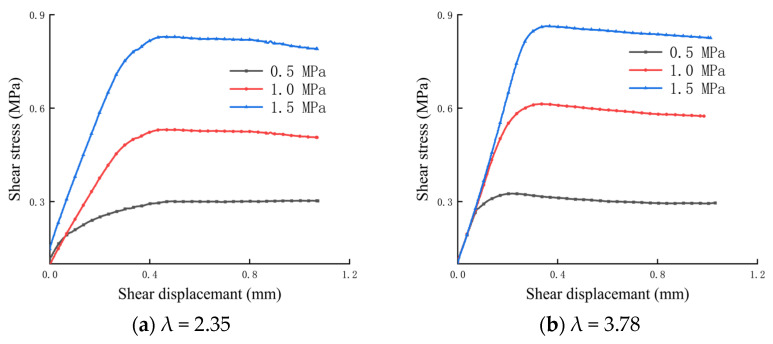

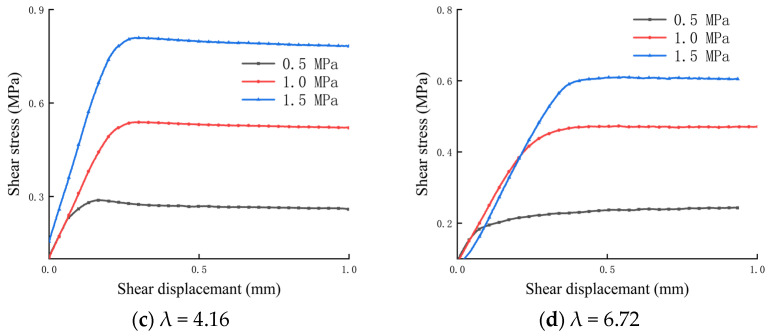
Shear stress–shear displacement curves of non–anchoring rock joint with different λ values.

Before carrying out the shear test of the bolted anisotropic rock joint, the shear stress–shear displacement curves of the non-bolted anisotropic rock joint with different λ values are shown in Figure 5. As can be seen from Figure 5, the shear stress–shear displacement curves of the unanchored rock joint under different λ values have the same variation trend, showing an approximately linear increase at first and a trend of smoothing after reaching the peak value. The shear strength increases with the increase in normal stress for the non-bolted joint with different λ values. Similar to when λ is 3.78, the peak shear strength increases from 0.325 MPa to 0.860 MPa when the normal stress increases from 0.5 MPa to 1.5 MPa.

**Figure 6:**
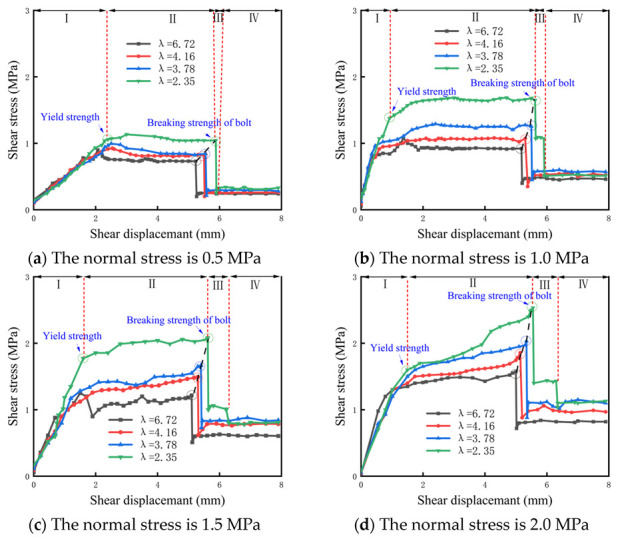
Shear stress – shear displacement curves of bolted anisotropic rock joint under different normal stress conditions.

The shear stress–shear displacement curves of the bolted rock joint with different λ values under different normal stresses are shown in Figure 6. The shear stress–shear displacement curves with different λ values have roughly the same trend, and the curves can be roughly divided into four stages: initial linear elastic stage, pre-breaking stage, breaking stage and residual stage, as shown in Figure 6:

Initial linear elastic stage (Section I): In this stage, the initial shear stress reaches the yield strength. At the same time, the curve basically presents an increasing linear trend, and the shear stress increases rapidly with the increase in shear displacement. This is because the anchor bolt has already played a role in the 0–2 mm shear displacement, indicating that the “pin” function of the anchor bolt is significant. At this time, the shear stress is mainly provided by the “pin” action of the bolt body, the friction between the rock joint and the extrusion pressure between the bolt, the anchorage agent and the rock mass. At this stage, the slope of the curve under the same normal stress and different λ values is basically the same, which indicates that there is little difference in shear stiffness. Under relatively small normal stress, such as 0.5 MPa, the yield strength is the peak value of the curve, and the peak value decreases with the increase in the normal stress and λ value. Taking the condition of 0.5 MPa normal stress as an example, when λ = 2.35, 3.78, 4.16 and 5.72, the peak values were 1.134, 0.9948, 0.9268 and 0.8047 MPa, respectively.

Pre-breaking stage of bolt (Section Ⅱ): In this stage, from the yield strength to the breaking strength of the bolt, the slope of the curve in this stage decreases, and with the increase in shear displacement, the increased rate of shear stress slows down. It can also be seen from Figure 5 that the normal stress has a significant influence on the shear stress–shear displacement curve at this stage, and the shear stress–shear displacement curve shows different trends under different normal stresses, but the λ value has little influence on the curve at this stage. On the whole, when the normal stress is 0.5 MPa, the curve shows a softening stage, and the shear stress decreases with the increase in shear displacement. When the normal stress is 1 MPa, the curve shows slight fluctuation, and the trend is a slight rise or fall. When the normal stress is greater than 1 MPa, the curve shows a hardening stage. At this time, the shear stress increases with the increase in shear displacement and reaches the peak value at the first breaking point of the bolt; that is, the breaking strength of the bolt is the peak strength of the curve. This is because the increase in normal stress increases the axial force of the bolt, and the shear stress increment provided by the axial force of the bolt can offset or even exceed the shear stress reduction caused by the failure of the rock joint. In addition, it can be seen that the shear stress of a broken bolt has a direct relationship with λ value and normal stress, and the shear stress increases with the increase in λ value and normal stress.

Rock bolt breaking stage (Section Ⅲ): In this stage, the shear stress–shear displacement curve first shows an obvious drop from the breaking of the bolt. This is because when the bolt breaks after reaching the deformation limit, the bolt no longer provides shear strength, and the curve shows a sudden drop. Moreover, the clear sound of a “bang” rock bolt breaking can be heard in this stage. The shear stress quickly drops to the minimum value and then rises to a certain value with a small amplitude.

Residual stage (Section Ⅳ): This stage begins after the curve falls and bounces back, and then the curve shows some slight fluctuation or rises or falls. The slight fluctuations in the curve are roughly the same as, or slightly higher than, the unanchored curve. In addition, if the λ value is small, the curve appears to have a second drop, the curve trend of the second fall behind and no anchor state is basically the same. This is because the first broken bolt exposed protruding in the shear process will be subjected to rock joint resistance. With the increase in shear displacement, exposed protruding further deformation, and in the shear displacement reaching a certain value, the protrusion breaks, with a dull “bang” sound when broken, and the curve shows a secondary drop.

In addition, it can be seen that whether it is in the initial linear elastic stage to achieve the yield strength or bolt breaking strength to the stage before the bolt breaking is related to the size of the λ value and decreases with the increase in the λ value, this is because, with the increase in the λ value, the gap between the strength of the footwall rock on the structural surface widened. In the same case of footwall coal, the greater the strength of the hanging rock, the shear provided by the axial force of the bolt gradually decreases, while the shear provided by transverse shear force gradually increases, but the total shear of the bolt gradually decreases [25].

### 3.2. Analysis of Shear Strength Characteristics

**Figure 7:**
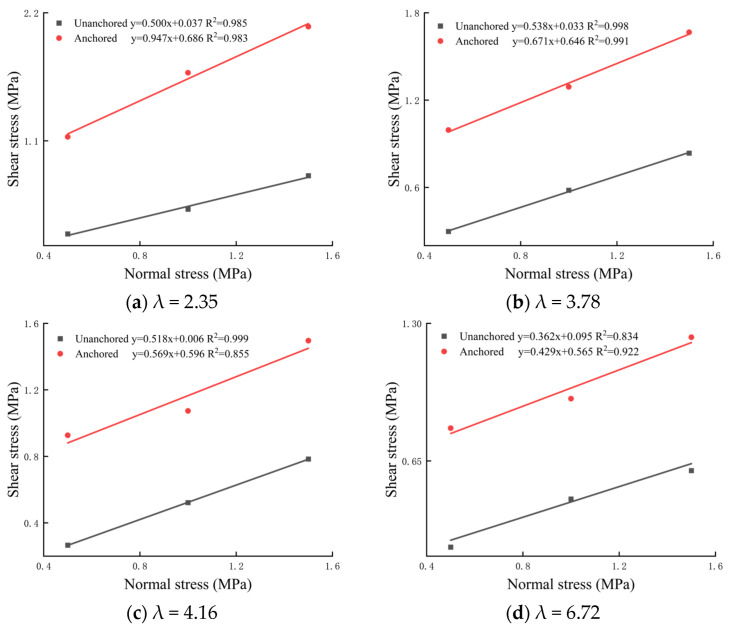
Variation characteristics of peak shear strength under different λ values of bolted and non-bolted.

The shear strength of the bolted rock joint is the main parameter to describe the shear performance of the bolted rock joint, which has important significance in the design of bolted support. To study the shear strength of the bolted rock joint with different λ values and the enhancement effect of bolted on the shear strength of the rock joint, the peak strength of unanchored and bolted anisotropic rock joint under normal stress of 0.5, 1 and 1.5 MPa was extracted, and the results were compared and analyzed. The variation characteristics of peak shear strength under anchoring and no anchoring conditions are shown in Figure 7. By fitting the experimental data in the figure, the relationship between shear strength and normal stress of anisotropic rock joints under bolted and non-bolted conditions is obtained. It can be seen that the shear strength of the bolted anisotropic rock joint and non-bolted anisotropic rock joint conforms to the Mohr–Coulomb strength criterion, i.e.,

(1) τ=c+σtanφ

In Equation (1), τ is shear stress, c is rock cohesion, σ is normal stress, and φ is internal friction angle.

It can be seen from the figure that the slope and longitudinal intercept of the fitting curve of the bolted anisotropic rock joint are both larger than that of the fitting curve without anchors, indicating that the bolt contributes significantly to the shear strength of the anisotropic rock joint and can effectively increase the cohesion and internal friction angle of the joint, which is the same as that of the isotropic rock joint [26]. At the same time, it can be found from the comparison of the curve of different λ values of anchoring rock joint in Figure 7 with the increase in λ value that the slope of the curve of the bolted anisotropic rock joint decreases significantly. It can be seen that the larger the λ value, the smaller the internal friction angle of the bolted anisotropic rock joint.

**Figure 8:**
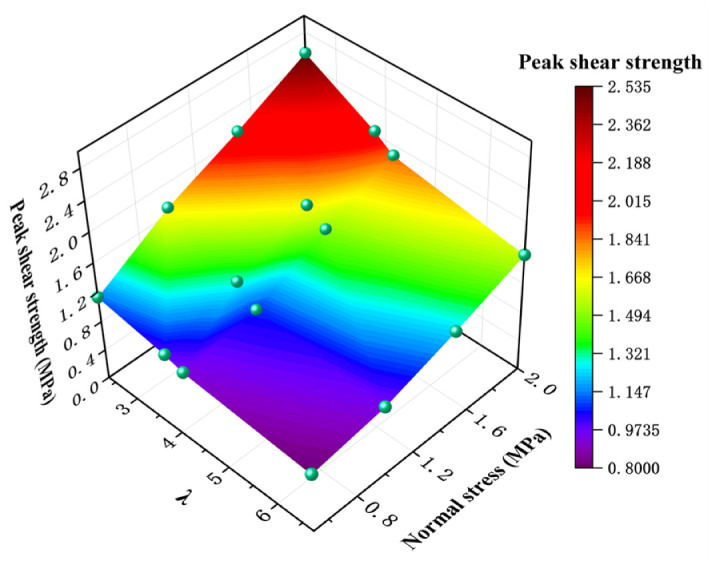
Relationship between peak shear strength and λ under different normal stress conditions.

Peak shear strength is an important representation of rock joint shear mechanical properties. The peak shear strength characteristics of different λ values are shown in the figure below. It can be seen from Figure 8 that under the same normal stress condition, the peak shear strength decreases with the increase in λ value. This indicates that the greater the strength difference between the two sides of the rock joint, the worse the shear capacity of the rock joint [27,28]. With the same λ value, the peak shear strength increases with the increase in normal stress, and the normal stress can effectively improve the shear strength of the rock joint. However, when the λ value is larger, the normal stress has no obvious effect on the shear strength of the rock joint.

**Figure 9:**
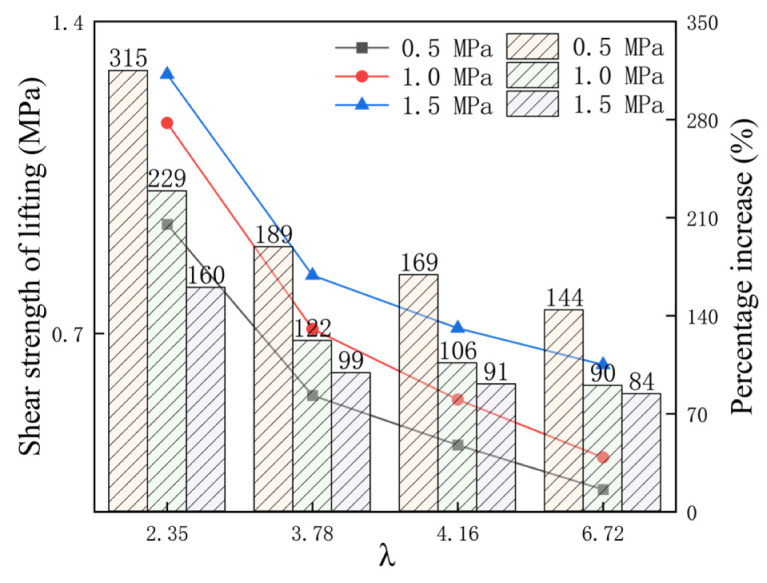
The shear strength increased by different λ values.

In order to more directly study the lifting effect of different λ values on anisotropic rock joint, the shear strength (the difference between the peak shear strength under anchoring and that without anchoring) increased by different λ values of the anisotropic rock joint under conditions of 0.5 MPa, 1 MPa and 1.5 MPa, and the percentage of lifting strength were extracted as shown in the figure below. The percentage of lifting strength is (shear strength of anchorage–shear strength without anchorage)/shear strength without anchorage × 100%. It can also be seen from Figure 9 that with the increase in λ value, the shear strength increased by the bolted rock joint under different normal stress conditions gradually decreases. Under the condition of 1 MPa normal stress, when the λ value increases from 2.35 to 6.72, the shear strength increased by 1.1724, 0.7105, 0.5518 and 0.4221 MPa, respectively. Under the same λ, the increased shear strength percentage decreases with the increase in normal stress. When λ = 2.36 and the normal stress increases from 0.5 MPa to 1.5 MPa, the increased shear strength percentage decreases from 315% to 160%. Under different λ, the increased shear strength is different. With the increase in λ, the increased shear strength percentage gradually decreases.

### 3.3. Analysis of Shear Failure Characteristics of Rock Joint

**Figure 10:**
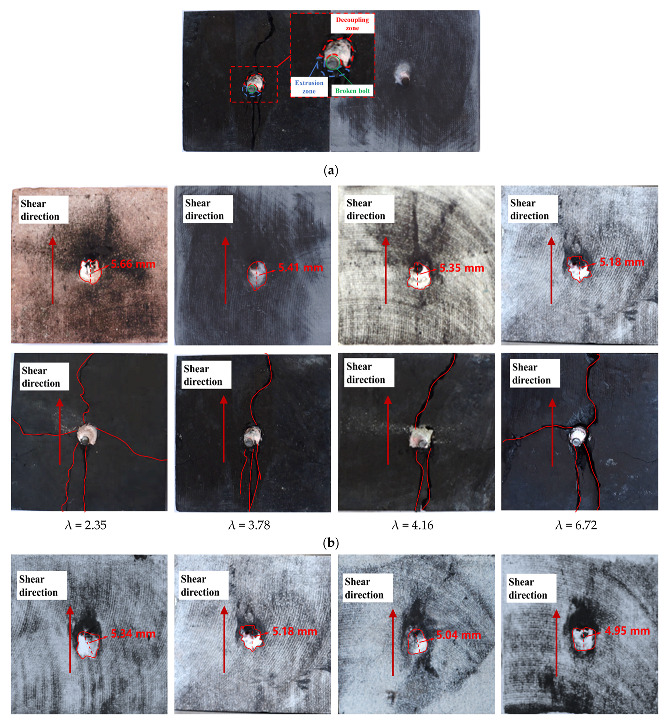

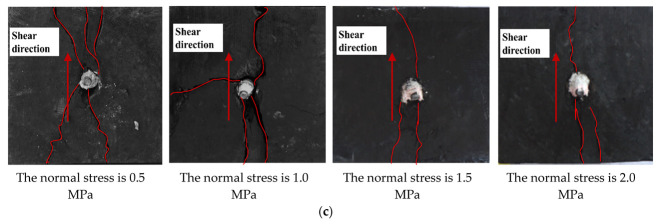
Shear failure characteristics of bolted anisotropic rock joint. (**a**) Structural surface failure and rock bolt failure. (**b**) Surface failure characteristics of the bolted anisotropic rock joint under 1 MPa normal stress (above is the surface of sandstone, and below is the surface of coal). (**c**) Surface failure characteristics of bolted anisotropic rock joint under different normal stress conditions (λ = 6.72, above is the surface of sandstone, and below is the surface of coal).

When the bolt plays the role of transverse shear in the bolted rock mass, it is bound to produce a certain transverse deformation due to the comprehensive action of external load and surrounding rock extrusion. To further analyze the shear failure characteristics of the bolt in the bolted anisotropic rock joint, it is very necessary to analyze the failure characteristics of the rock joint and the bolt. Figure 10a shows the failure characteristics of the rock joint and bolt breaking, and Figure 10b shows the surface failure characteristics of the bolted anisotropic rock joint with different λ values under 1 MPa normal stress. Above is the surface of sandstone, and below is the surface of coal; Figure 10c shows the surface failure characteristics of bolted anisotropic rock joints under different normal stresses. Above is the surface of sandstone, and below is the surface of coal. The injection range in the figure is the failure area of the rock joint, and the red line in the figure is the joint fracture path.

As can be seen from Figure 10, under different λ values, through-splitting failure occurs along the shear direction on the footwall coal structural surface, and exposed protrusions of the broken bolt are left on the surface of the coal structural surface, with varying lengths of protrusions. With the increase in the λ value, the length of protrusions gradually decreases. It can also be seen from Figure 10a that there are extrusion zone and decoupling zone [9] on both sides of the rock joint anchor bolt. The extrusion zone is that under the action of shear force, the anchor bolt and surrounding rock extrude each other, resulting in the local rock mass and anchorage agent being extruded and broken and obvious pink anchoring agent powder appearing. In the decoupling area, the bolt and the anchor agent are separated due to the tension under the action of shear force. In addition, the bolt also broke and lost its shear performance.

At the same time, it can be seen from Figure 10b,c that the hanging wall rock structure presents obvious elliptical failure near the face wall. The long axis direction of the ellipse is the shear direction, and the value marked in the figure is the size of the long axis of the ellipse. On the one hand, the reason for the elliptical failure is that the bolt compresses the pore wall in the shear process, which causes the extrusion destruction of the anchorage agent and rock mass at the rock joint. The residual pink powder of the anchorage agent on the surface of the footwall coal pore wall also verifies this reason. On the other hand, the shear process continues after the breaking of the bolt, and the protruding bolt of the rock joint continues to slide along with the shear process. In the sliding process, the rock on the hanging wall continues to be damaged until the second failure of the bolt or the end of the shear occurs. However, compared with the failure reasons mentioned in the former, the latter causes a lower degree of damage. It can be seen from Figure 10b that when λ value is 2.35, 3.78, 4.16 and 6.72, the long axis lengths of the failure ellipse caused by bolt extrusion and protrusion sliding are 5.66, 5.41, 5.35 and 5.18 mm, respectively. It can be seen that the long axis lengths of the failure ellipse decrease with the increase in λ value. This is because with the increase in λ value, the strength of the hanging wall rock gradually increases, and the damage degree of the bolt to the relatively small strength rocks such as red sandstone is greater, and the damage degree of the larger strength rocks such as white sandstone is less. Another reason is that with the increase in rock strength, the transverse deformation of the bolt becomes smaller, and the length of the exposed projection of the bolt becomes smaller. As a result, the friction degree of exposed projection on rock decreases, and the damage range decreases. As can be seen from Figure 10c, when footwall rock strength is fixed on the rock joint, the difference of normal stress also affects the elliptic failure range near the face wall of the rock mass structure, and the long axis of the ellipse gradually decreases with the increase in normal stress. This is because the increase in normal stress makes the axial force on the bolt increase, the transverse deformation of the bolt is limited, and the damage degree to the surrounding rock is reduced.

### 3.4. Shear Deformation Fracture Characteristics of Bolt

**Figure 11:**
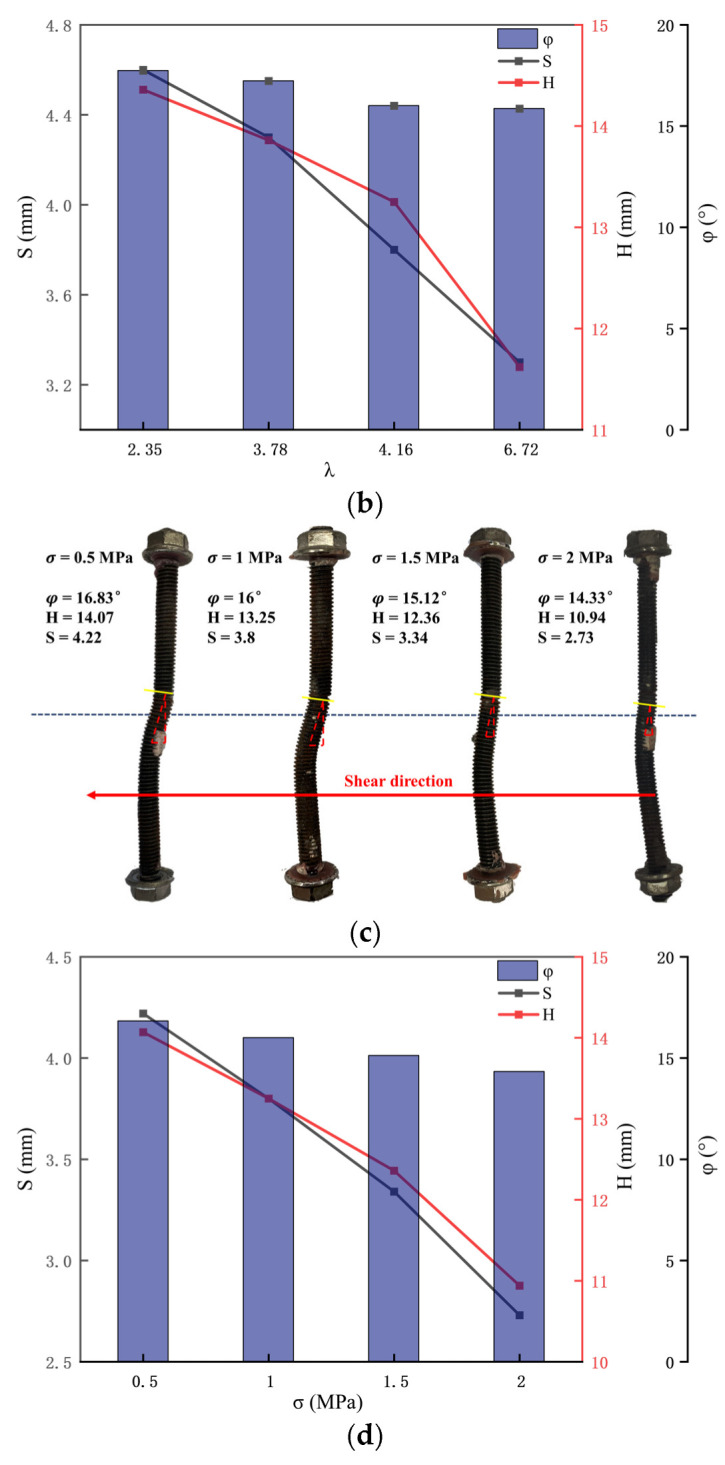
Shear deformation fracture diagram of bolt. (**a**) Shear deformation and breaking characteristics of anchor bolts in anisotropic rock joints under 1 MPa normal stress. (**b**) The deformation of bolt changes under 1 MPa normal stress. (**c**) Shear deformation breaking characteristics of anchor bolts in anisotropic rock joints with different normal stresses (λ = 4.16). (**d**)The deformation of bolt changes with different normal stresses (λ = 4.16).

The enhancement of shear strength characteristics of the bolted anisotropic rock joint of the bolt is premised on the deformation of the bolt in the shear process. Therefore, the analysis of the deformation and failure forms of the bolt after the shear test can better explain the anchoring mechanism of the bolt [29]. Shear deformation and breaking characteristics of the anchor bolt under 1 MPa normal stress are shown in Figure 11. The yellow line segment in the figure marks the broken section of the anchor bolt, φ, S and H are shear angles, respectively, and the horizontal and vertical components of the length of the deformed part of the anchor bolt.

As can be seen from Figure 11, the bolts with different λ values of bolted anisotropic rock joints all show the deformation characteristics of the “S” shape after shear breaking. Moreover, due to the different strengths of the upper and footer rocks, the deformation ranges of the bolts on both sides of the rock joint are not the same, so the plastic hinges formed on both sides of the rock joint are not symmetrical, which is different from the same type of bolted rock joint. At the same time, it can be observed that the length of the plastic hinge on both sides of the rock joint is about two to three times the diameter of the anchor rod, which is consistent with the existing theory [30]. It can be seen from Figure 11a,b, the deformation range of the bolt gradually decreases with the increase in λ value. When λ value is 2.35, 3.78, 4.16 and 6.72, the horizontal component S of the deformation part of the bolt is 4.6, 4.3, 3.8 and 3.3 mm, respectively, showing a decreasing trend. H is 14.36, 13.86, 13.25 and 11.62 mm, respectively, which also shows a decreasing trend. This is mainly because the strength of hanging wall rock increases, the constraint effect of surrounding rock on the anchor bolt is enhanced, and the deformation of the anchor bolt becomes smaller. It can also be found from Figure 11c,d that when the λ value remains unchanged, the deformation range of the bolt decreases gradually with the increase in normal stress. When the normal stress is 0.5, 1,1.5 and 2 MPa, the horizontal component S of the deformation part of the bolt is 4.22, 3.8, 3.34 and 2.73 mm, respectively. The vertical component H of the deformation part of the bolt is 14.07, 13.25, 12.36 and 10.94 mm, respectively. Both S and H showed a decreasing trend.

**Figure 12:**
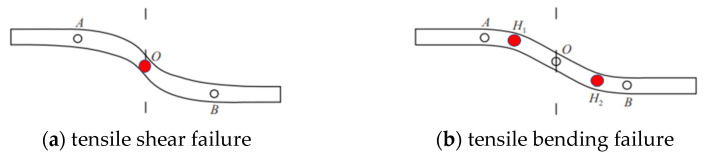
Failure model of structural face bolt.

Liu et al. [31] believe that there are two main failure models of the bolt. One is the tensile shear failure of the bolt under the combined action of axial force and shear force, which usually occurs at the rock joint, as shown in Figure 12a. The bending moment on the cross-section of the bolt at O point is 0, and the bolt is in a tension shear state. The other is under the action of tensile shear load, and the plastic hinge appears at 2–4 times the diameter of the bolt on both sides of the rock joint. With the further increase in shear load, the anti-symmetric yield points H_1_ and H_2_ along the joint surface form a plastic hinge. Once the anti-symmetric plastic hinge is formed, the shear load that the bolt can bear does not increase. Since the plastic hinge is located at the point of maximum bending moment, the shear force on the cross-section of the anchor bolt at this point is 0, as shown in Figure 12b. The location of the broken bolt is the main way to distinguish the two kinds of failure. As can be seen from the figure, when λ = 2.35, the bolt is broken into three segments, which corresponds to the second drop of the CD segment curve mentioned above. When λ = 3.78, 4.16 and 6.72, the bolt is broken into two segments, and no matter what the value of λ, the bolt breaking position is at the plastic hinge point, indicating that all the bolts have tensile bending failure. This is because the footwall of the bolt is exposed after it is broken under a shear load. Under the action of the normal stress of the rock joint, on the one hand, the bolt protrusion is partially pressed into the rock block, limiting the slide of the bolt. On the other hand, it causes a large tensile stress on one side of the plastic hinge of the bolt, resulting in the bending failure of the bolt under the comprehensive action of the two. The smaller the λ value is, the farther the breaking position of the bolt is from the rock joint, indicating that the increase in λ value limits the development of plastic hinges away from the rock joint.

### 3.5. Deformation Analysis of Shear Bolt Mechanical Model

**Figure 13:**
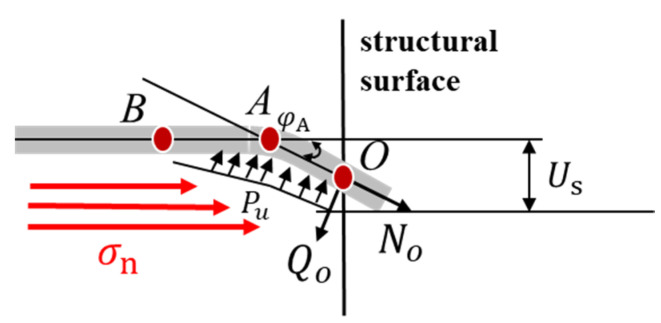
Bolt deformation diagram.

Under shear action, apart from axial tensile deformation, the anchor bolt appears to be bending deformation within a certain range near the joint surface, and the rotation effect is caused when the section reaches complete plasticity at the point of maximum bending moment, which is usually referred to as plastic hinge [16], as shown in Figure 13. Point A is the plastic hinge point, the shear force at this point is 0, and the bending moment reaches the maximum. $\sigma_{n}$ is the normal stress, and $U_{s}$ is shear deformation displacement of bolt. The axial force and shear force at point O are N_O_ and Q_O,_ respectively. The reaction force of slurry on the bolt is Pu. With the shear bending of the bolt, the normal stress is exerted on the OA section of the bolt through the rock mass, and $\varphi_{A}$ is the rotation angle of the bolt at O point.

**Figure 14:**
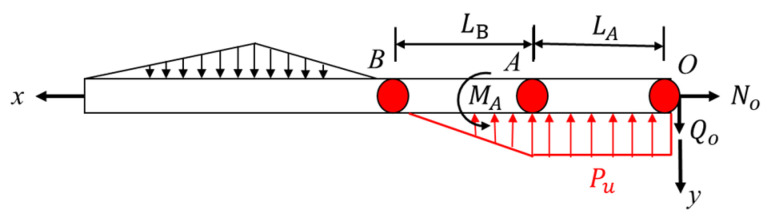
Stress analysis diagram of bolt.

The formation of a plastic hinge is related to the establishment of an anchor bolt. Scholars at home and abroad usually adopt the classical beam theory of elastic foundation to solve the shear force of anchor bolts. In the beam theory of elastic foundation, the anchor bolt is regarded as a semi-infinite beam on an elastic foundation, and surrounding concrete and rock mass are regarded as an elastic foundation [25]. In the shearing process, rock mass and anchor bolt squeeze each other, resulting in the reaction force of rock mass on the compressive side of the anchor bolt. Since the footwall rocks of the rock joint are all coal, the main difference lies in the hanging wall rocks. The stress analysis of the anchor bolt on the rock joint is carried out, and its stress schematic diagram is shown in Figure 14.

In the shear process of bolted joint, two singular points are formed on the bolt: the intersection point O between the bolt and the joint, the bending moment of the point is 0, and the shear force is maximum. Point A is the plastic hinge point, the shear force at this point is 0, and the bending moment reaches the maximum. The axial force and shear force at point O are N0 and Q0, respectively, set at $\bar{AO}$ segment of length $L_{A}$, and $\bar{AB}$ segment length of $L_{B}$. By force analysis of section AB, it can be obtained [20]:

(2) $$\sum F_{y}=0\Rightarrow L_{A}=\frac{Q_{0}}{P_{u}}$$

By bending equilibrium at point A, you obtain:

(3) $$\sum M=0\Rightarrow M_{A}=\frac{Q_{0}{}^{2}}{2P_{u}}$$

**Figure 15:**
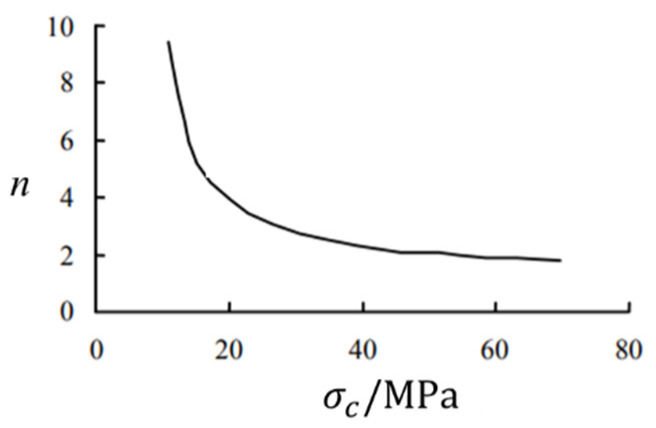
n value under different compressive strength of surrounding rock.

In the formula, $F_{y}$ is the transverse force on the bolt $\bar{AO}$ section; $M_{A}$ is the bending moment of the bolt at point A; $P_{u}=n\sigma_{c}D$ for $\bar{AO}$ bolt pressure side rock mass along the length direction of the reaction degree; D is the bolt diameter; $\sigma_{c}$ is the compressive strength of the surrounding rock or anchoring agent; and n is the force coefficient for experience value, usually between 1 and 15 values [17,32,33]. n is related to the compressive strength of rock and gradually increases with the decrease in compressive strength, and the smaller the strength, the greater the increase in n, as shown in Figure 15.

As can be seen from the figure above, with the increase in the compressive strength of the surrounding rock, the ultimate reaction on the compressive side of the anchor rod during bending increases; namely, $P_{u}$ increases [25].

**Figure 16:**
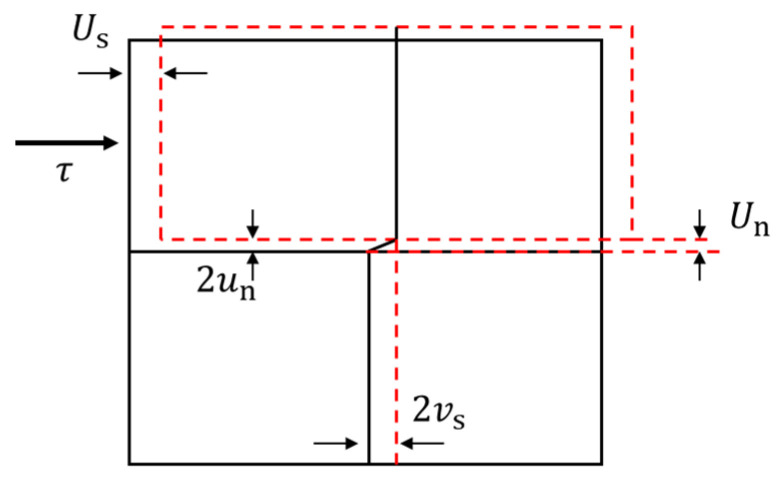
Schematic diagram of stress and deformation of bolted anisotropic rock joint.

It is assumed that the shear displacement of rock joint is $U_{s}$, the normal displacement is $U_{n}$, and the normal displacement is mainly generated by dilatancy. The schematic diagram is shown in Figure 15. According to geometric relations, the initial axial deformation and transverse deformation components of the anchor bolt on one side of the rock joint can be deduced from the shear displacement and normal displacement of the rock joint, namely, $u_{n}$ and $v_{s}$ in Figure 16.

(4) $$u_{n}=0.5U_{n}$$

(5) $$v_{s}=0.5U_{s}$$

It is assumed that when shear sliding occurs on the rock joint, the axial displacement and lateral displacement of the anchor bolt on one side of the rock joint are, respectively [34].

(6) $$u\left(x\right)=-u_{n}\left(1-\frac{2x}{3\pi l_{0}}\right)$$

(7) $$v\left(x\right)=v_{s}e^{-\frac{x}{l_{0}}}\cos\left(\frac{x}{l_{0}}\right)$$

According to Formulas (6) and (7), the strain energy V and external potential W of the anchor bolt on one side of the rock joint can be obtained, respectively. According to the standing value principle of potential energy, the real displacement field satisfying the minimum potential energy can be obtained [35]:

(8) $$u_{n}=\frac{24N_{0}Q_{0}}{EP_{u}\pi D^{2}}$$

(9) $$v_{s}=\frac{8192Q_{0}{}^{4}b}{EP_{u}{}^{3}\pi^{4}D^{4}}$$

where b = 0.27.

According to Equations (8) and (9), with the increase in λ value, the ultimate reaction of rock mass on the compressive side of the bolt increases, and the axial and transverse deformation components of the bolt become smaller, which is consistent with the deformation characteristics of the bolt in the previous section.

### 3.6. Analysis of Fracture Shear Displacement Characteristics of Bolt

The peak shear displacement of rock joints has an important application in the practical engineering of rock mass and is also an important parameter to describe the pre-peak shear stress–shear displacement curve [13]. Similarly, it is important to determine the breaking displacement of bolts to ensure that the shear deformation of the rock joint is in a controllable range.

**Figure 17:**
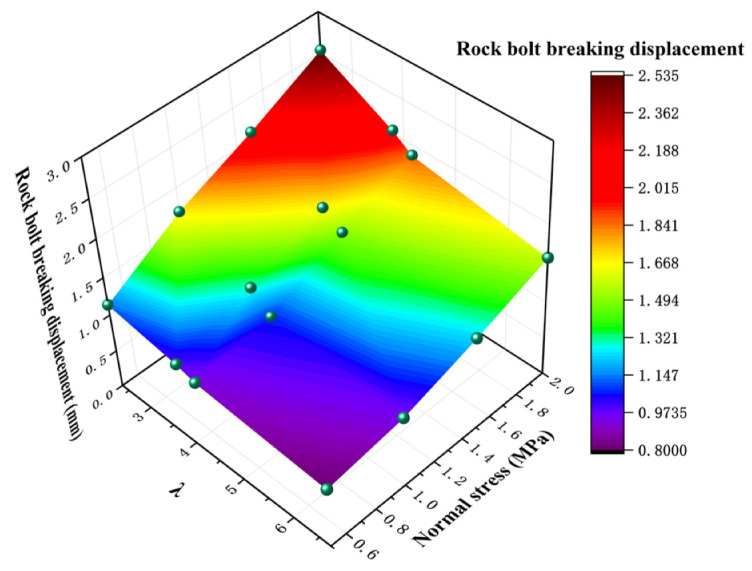
Fracture shear displacement curves of bolt under different normal stress conditions.

The fracture shear displacement characteristic curves of bolted rock joints with different λ values under different normal stress conditions are shown in Figure 17. It can be seen that with the increase in normal stress, the fracture displacement of the anchor bolt decreases continuously. The possible reason for this phenomenon is that the increase in normal stress leads to the more intense extrusion effect of rock mass near the joint on the anchor bolt. Under high normal stress, the plastic deformation of the bolt is allowed to be smaller so that the bolt breakage occurs earlier in the shear process [36]. The normal external load causes the rocks on both sides of the rock joint to squeeze each other, resulting in the strengthening of the bearing capacity of rock materials and the increase in the reaction force on the bolt. Under the same shear displacement condition, the greater the reaction force on the bolt, the greater the shear force on the bolt, and the axial force on the bolt decreases relatively, but the change range is not large. The larger the shear force on the bolt, the larger the bending moment at the plastic hinge, and the shear displacement required to reach the maximum breaking bending moment of the bolt decreases [20]. At the same time, it can be seen from Figure 12 that the breaking shear displacement of the bolt decreases gradually with the increase in λ value. By taking the condition of 1 MPa normal stress as an example, when λ = 2.35, 3.78, 4.16 and 6.72, the breaking shear displacement of the bolt is 2.73, 5.5, 5.38 and 5.18 mm, respectively. This is because the footwall coal remains unchanged, the strength of the hanging wall rock gradually increases, and the ultimate compressive force of the hanging wall anchor bolt increases when bending, resulting in the transverse shear force of the anchor bolt increases, while the axial force decreases correspondingly because the bending yield strength of the anchor bolt has been reached under the small shear displacement, and then the breaking occurs. Moreover, combined with the above, it can be found that the breaking displacement of the bolt is directly proportional to the transverse deformation of the bolt.

## 4. Conclusions

In this paper, a series of laboratory shear tests are carried out on the bolted anisotropic rock joint, and the characteristics of shear stress–shear displacement curve, shear strength, failure characteristics of rock joint and failure characteristics of the bolt are discussed. From the test results, the main conclusions can be drawn. In future studies, we will further deepen the study of shear tests of anchorage structural planes under different rock combinations.

(1) The shear stress–shear displacement curve of the bolted anisotropic rock joint can be divided into four stages: initial linear elastic stage, bolt breaking stage, bolt breaking stage and residual strength stage. In the initial linear elastic stage, the shear stress increases approximately linearly with the increase in shear displacement. The shear stress shows a different trend with the increase in shear displacement under different normal stress. In the rock bolt breaking stage, the rock bolt breaks when it reaches the bending limit, and the stress drops on the curve. In the residual strength stage, the shear strength in this stage is the same or slightly higher than that in the unanchored state.
(2) The shear strength of bolted anisotropic rock joint and non-bolted anisotropic rock joint conforms to Mohr–Coulomb strength criterion. The shear strength of the rock joint is enhanced by increasing the cohesion and internal friction angle of the rock joint. With the increase in λ value, the slope of the curve of the bolted anisotropic rock joint decreases obviously. It can be seen that the larger the λ value is, the smaller the friction angle of the bolted anisotropic rock joint is. Under the same normal stress condition, the peak shear strength decreases with increasing λ value. This indicates that the greater the strength difference between the two sides of the rock joint, the worse the shear capacity of the rock joint.
(3) The elliptic failure occurs on the surface of the rock joint, and the length of the major axis of the ellipse decreases with the increase in λ value and normal stress. On the one hand, the cause of elliptical failure is that the bolt compresses the pore wall in the shear process, resulting in the extrusion destruction of the anchoring agent and rock mass at the rock joint. The residual pink anchoring agent powder on the surface of the footwall coal pore wall also verifies this reason. On the other hand, the shear process continues after the breaking of the bolt, and the protruding bolt of the rock joint continues to slide along with the shear process. In the sliding process, the rock on the hanging wall continues to be damaged until the second failure of the bolt or the end of the shear occurs. However, compared with the failure reasons mentioned in the former, the latter causes a lower degree of damage.
(4) The bolts with different λ values show the deformation characteristics of the “Z” shape after a shear fracture. At the same time, the deformation range of the bolts on both sides of the rock joint is not the same because of the different strengths of the rock in the upper and footwall. The horizontal and vertical components of bolt deformation decrease with the increase in λ value and normal stress, and the same conclusion is obtained by theoretical analysis.
(5) The breaking shear displacement of the bolt increases with the increase in normal stress and decreases with the increase in λ value. When λ = 2.35, 3.78, 4.16 and 6.72, the breaking shear displacement of the bolt is 2.73, 5.5, 5.38 and 5.18 mm, respectively. This is because the footwall coal remains unchanged, the strength of the hanging wall rock gradually increases, and the ultimate compressive force of the hanging wall anchor bolt increases when bending, resulting in the transverse shear force of the anchor bolt increasing. Moreover, the axial force decreases correspondingly because the bending yield strength of the anchor bolt has been reached under the small shear displacement, and then the breaking occurs.

## Figures and Tables

**Table 1:** Anchor bolt and anchor agent basic performance parameter table.

Anchorage Agent: Planting Reinforcement Adhesive		Anchor Bolt: Carbon Steel	
Temperature	25 °C	Diameter	6 mm
Setting time	48 h	Length	100 mm
Tensile strength	50 MPa	Tensile strength	800 MPa
Elasticity modulus	3980 MPa	Yield strength	640 MPa
Flexure strength	76.3 MPa	Elongation	12%
Elongation	1.3%		

**Table 2:** The strength of the specimen material.

Title	Uniaxial Compressive Strength	Tensile Strength
White sandstone	86 MPa	6.45 MPa
Green sandstone	53.17 MPa	4 MPa
Black sandstone	48.33 MPa	3.28 MPa
Red sandstone	30 MPa	2.77 MPa
Coal	12.78 MPa	1.4 MPa

## Data Availability

Data associated with this research are available and can be obtained by contacting the corresponding author upon reasonable request.

## References

[1] Ban L.R., Qi C.Z., Shan R.L., Tao Z.G., Xia C., Jiang K. (2018). Pre-peak shear constitutive model considering the softening shear stiffness and its influencing factors. J. China Coal Soc..

[2] Wang C.S., Liu R.C., Jiang Y.J., Wang G., Luan H.J. (2022). Effect of shear-induced contact area and aperture variations on nonlinear flow behaviors in fractal rock fractures. J. Rock Mech. Geotech. Eng..

[3] Yang T.H., Wang P.T., Xu T., Yu Q.L., Zhang P.H., Shi W.H., Hu G.J. (2015). Anisotropic characteristics of jointed rock mass: A case study at Shirengou iron ore mine in China. Tunn. Undergr. Space Technol..

[4] Liu X.R., Xu B., Zhou X.H., Yi L., Zeng X., Wang J.W. (2021). Investigation on the macro-meso shear mechanical properties of soft-hard interbedded rock discontinuity. J. China Coal Soc..

[5] Luan H.J., Cao Y.W., Jiang Y.J., Guan Y.T., Li C.P., Zhang S.H., Liu J.R. (2022). Implementation of tension-shear coupling failure mode of rock bolts in FLAC3D and its application. J. Min. Strat. Control Eng..

[6] Wang Y.Y., Gong B., Zhang Y.J., Yang X.Y., Tang C.A. (2022). Progressive fracture behavior and acoustic emission release of CJBs affected by joint distance ratio. Mathematics.

[7] Gong B., Wang Y.Y., Zhao T., Tang C.A., Yang X.Y., Chen T.T. (2022). AE energy evolution during CJB fracture affected by rock heterogeneity and column irregularity under lateral pressure. Geomat. Nat. Hazards Risk.

[8] Wang Y.Y., Gong B., Tang C.A. (2022). Numerical investigation on anisotropy and shape effect of mechanical properties of columnar jointed basalts containing transverse joints. Rock Mech. Rock Eng..

[9] Tao Z.G., Ren S.L., He M.C., Xia M. (2022). Static tensile and bolting shear mechanical properties of micro-NPR bolt steel in underground engineering. J. China Coal Soc..

[10] Cheng G.T., Liu C.X., Zhang J., Zhang N., Cui G.J. (2022). Shear behaviour of reproduced rough and bolted marble joints using the 3D carving technique. Arab. J. Geosci..

[11] He M.C., Ren S.L., Guo L.J., Lin W.J., Zhang T.W., Tao Z.G. (2022). Experimental study on influence of host rock strength on shear performance of micro-npr steel bolted rock joints. Int. J. Rock Mech. Min..

[12] Jiang Y.J., Zhang S.H., Luan H.J., Chen L.J., Zhang G.C., Wang C.S. (2021). Numerical Investigation on the Effect of Cyclic Loading on Macro-meso Shear Characteristics of Rock Joints. J. China Coal Soc..

[13] Jiang Y.J., Zhang S.H., Luan H.J., Wang C.S., Wen Z.J., Wang D., Han W. (2021). Experimental study on shear characteristics of bolted rock joints under constant normal stiffness boundary conditions. Chin. J. Rock Mech. Eng..

[14] Srivastava L.P., Singh M. (2015). Effect of fully grouted passive bolts on joint shear strength parameters in a blocky mass. Rock Mech. Rock Eng..

[15] Wang G., Zhang Y.Z., Jiang Y.J., Liu P.X., Guo Y.S., Liu J.K., Ma M., Wang K., Wang S.G. (2018). Shear Behaviour and Acoustic Emission Characteristics of Bolted Rock Joints with Different Roughnesses. Rock Mech. Rock Eng..

[16] Zheng L.B., Wang L.Q., Zhu L.F., Jiang Y.F., Wang B. (2021). Experimental study on the effect of locking mode on shear characteristics of bolted rock joint. Rock Soil Mech..

[17] Spang K., Egger P. (1990). Action of fully-grouted bolts in jointed rock and factors of influence. Rock Mech. Rock Eng..

[18] Liu S.G., Huang Z.X., Zhang H. (2022). Study of Shearing Tests of Bolted Frictionless Joints with Different Anchorage Angles. Chin. J. Undergr. Space Eng..

[19] Zhang Y.H., Wang D.J., Tang H.M., Li C.D., Yi X.L. (2016). Study of shear strength characteristics of heterogeneous discontinuities using PFC2D simulation. Rock Soil Mech..

[20] Yang B.Y., Xiao M., Luo N., Wang X.W. (2019). Research on mechanical characteristic of anchor bolt in process of shearing. J. Huazhong Univ. Sci Tech..

[21] He J.M., Wu G. (1994). The criterion tor shear strength of discontinuties with different rock properties in rock mass. J. Chongqing Univ..

[22] Fang K., Wu Q., Wang J., Tan F.L. (2014). Research on Shear Characteristics and the Evolution Mechanism of Bedding Planes Between Two Different Rock Types Based on Particle Flow Code. J. Yangtze River Sci. Res. Inst..

[23] Del Greco O., Ferrero A.M., Oggeri C. (1993). Experimental and analytical interpretation of the behaviour of laboratory tests on composite specimens. Int. J. Rock Mech. Min. Sci..

[24] Cui G.J., Zhang C.Q., Liu L.P., Zhou H., Cheng G.T. (2018). Study of effect of shear velocity on mechanical characteristics of bolt-grout interface. Rock Soil Mech..

[25] Chen W.Q., Zhao Y.F., Zhou J.J. (2018). Shear resistance theory of bolt considering nonlinear behaviour of grout reaction force. Rock Soil Mech..

[26] Liu Q.S., Lei G.F., Peng X.X., Wei L. (2018). Shearing mechanical model and experimental verification of bolts in jointed rock mass. Chin. J. Geotech. Eng..

[27] Andres U., Timoshkin I., Jirestig J., Stallknecht H. (2001). Liberation of valuable inclusions in ores and slags by electrical pulses. Powder Technol..

[28] Fukushima K., Kabir M., Kanda K., Obara N., Fukuyama M., Otsuki A. (2022). Simulation of Electrical and Thermal Properties of Granite under the Application of Electrical Pulses Using Equivalent Circuit Models. Materials.

[29] Zhang W., Liu Q.S. (2014). Analysis of deformation characteristics of prestressed anchor bolt based on shear test. Rock Soil Mech..

[30] Ge X.R., Liu J.W. (1988). Study on shear behavior of anchorage joint surface. Chin. J. Geotech. Eng..

[31] Liu C.H., Li Y.Z. (2018). Research progress in bolting mechanism and theories of fully grouted bolts in jointed rock masses. Chin. J. Rock Mech. Eng..

[32] Ferreroq A.M. (1995). The shear strength of reinforced rock joints. Int. J. Rock Mech. Min. Sci. Geomech. Abstr..

[33] Holmberg M. (1991). The mechanical behavior of untensioned grouted rock bolts. Ph.D. Thesis.

[34] Pellet F., Egger P. (1996). Analytical model for the mechanical behaviour of bolted rock joints subjected to shearing. Rock Mech. Rock Eng..

[35] Yang S.L., Xu Y.W., Huang Q.P. (2004). Analysis on the bolt deformation as result of joint shear displacement. Chin. J. Rock Mech. Eng..

[36] Wang L.Q., Zhu L.F., Zheng L.B., Fan B.Q., Zhang Z.H., Chen H.J. (2021). Shear Test of Bolted Joint Rock Masses Considering Joint Roughness. China J. Highw. Transp..

